# Renal Function After CT-Guided Cryoablation of Small Renal Tumours in Patients with Solitary Kidney: An Analysis of European Multinational Prospective EuRECA Registry

**DOI:** 10.1007/s00270-023-03634-4

**Published:** 2024-01-25

**Authors:** Pia I. Pietersen, Sarah Stougaard, Francis X. Keeley, Brunolf Lagerveld, David Breen, Alexander King, Tommy K. Nielsen, Marco van Strijen, Julien Garnon, Des Alcorn, Eric de Kerviler, Patricia Zondervan, Tze M. Wah, Theresa Junker, Ole Graumann

**Affiliations:** 1 Department of Radiology, Odense University Hospital, Kløvervænget 37, 5000 Odense, Denmark; 2 UNIFY – Research and Innovation Unit of Radiology, University of Southern Denmark, Odense, Denmark; 3 Bristol Urological Institute, North Bristol NHS Trust, Bristol, UK; 4 Department of Urology, OLVG, Amsterdam, The Netherlands; 5 Department of Radiology, Southampton University Hospitals, Southampton, UK; 6 Department of Urology, Aarhus University Hospital, Aarhus, Denmark; 7 Department of Radiology, St Antonius Hospital, Nieuwegein, The Netherlands; 8 Department of Interventional Radiology, Nouvel Hôpital Civil, 67096 Strasbourg cedex, France; 9 Department of Interventional Radiology, Gartnavel General Hospital, Glasgow, UK; 10 Radiology Department, Saint-Louis Hospital, AP-HP, 1, Avenue Claude-Vellefaux, 75475 Paris cedex 10, France; 11 Department of Urology, Amsterdam UMC, University of Amsterdam, Amsterdam, The Netherlands; 12 Department of Diagnostic and Interventional Radiology, Institute of Oncology, Leeds Teaching Hospitals Trust, St. James’s University Hospital, Leeds, UK

**Keywords:** Cryoablation, Image-guided, Renal cell carcinoma, Renal masses, Renal function, Solitary kidney

## Abstract

**Purpose:**

Treatment of renal cell carcinoma (RCC) in patients with solitary kidneys remains challenging. The purpose of this multicentre cohort study was to explore how renal function is affected by percutaneous image-guided cryoablation in patients with solitary kidneys.

**Material and Methods:**

Data from the European Registry for Renal Cryoablation database were extracted on patients with RCC in solitary kidneys treated with image-guided, percutaneous cryoablation. Patients were excluded if they had multiple tumours, had received previous treatment of the tumour, or were treated with more than one cryoablation procedure. Pre- and post-treatment eGFR (within 3 months of the procedure) were compared.

**Results:**

Of 222 patients with solitary kidneys entered into the database, a total of 70 patients met inclusion criteria. The mean baseline eGFR was 55.8 ± 16.8 mL/min/1.73 m^2^, and the mean 3-month post-operative eGFR was 49.6 ± 16.5 mL/min/1.73 m^2^. Mean eGFR reduction was − 6.2 mL/min/1.73 m^2^ corresponding to 11.1% (*p* = 0.01). No patients changed chronic kidney disease group to severe or end-stage chronic kidney disease (stage IV or V). No patients required post-procedure dialysis.

**Conclusion:**

Image-guided renal cryoablation appears to be safe and effective for renal function preservation in patients with RCC in a solitary kidney. Following cryoablation, all patients had preservation of renal function without the need for dialysis or progression in chronic kidney disease stage despite the statistically significant reduction in eGFR.

**Level of Evidence 3:**

Observational study.

**Graphical Abstract:**

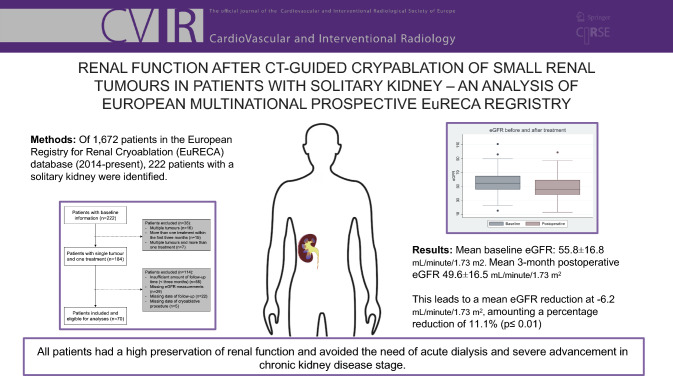

## Introduction

The incidence of renal cell carcinomas (RCC) continues to increase—especially in high-income countries [1]. Each year around 400,000 patients worldwide are diagnosed with RCC, and RCC is associated with 175,000 cancer deaths per year globally [2, 3].

According to the 2023 European Association of Urology (EAU) guidelines for kidney cancer, nephron-sparing surgery including open, laparoscopic, or robot-assisted partial nephrectomy remains the primary recommended treatment for small RCCs defined as T1 tumours [4]. However, within the last decade, the use of percutaneous, CT-guided cryoablation for T1 RCCs has been increasingly used and evaluated in many clinical studies [5–7]. Image-guided renal cryoablation has emerged as potentially less invasive and more nephron-sparing than surgical treatment for renal function preservation, leading to a shorter hospital stay and causing fewer complications. The long-term oncological outcomes are yet to be confirmed by randomized controlled trials; however, the emerging evidence is beginning to suggest that image-guided cryoablation could be comparable to surgical short- and long-term outcomes [7]. However, the debate is ongoing and most international guidelines still recommend laparoscopic- or robot-assisted partial nephrectomy over cryoablation for T1 RCCs [8].

Since 2017, the American Urological Association (AUA) and European Society for Medical Oncology (ESMO) guidelines have endorsed image-guided cryoablation to be considered for patients with comorbidities such as non-surgical candidates, previous major renal surgery, or patients with a solitary kidney [8, 9]. In the 2022 update presented at the annual AUA meeting, the AUA directly suggests that clinicians should consider ablative therapy for T1a solid tumours below 3 cm [10], especially in frail or non-surgical candidates, or in patients with solitary kidney and small RCCs. While still being debated, in these patients partial nephrectomy could carry a risk of compromising renal function due to the removal of functional renal parenchyma or prolonged ischaemic time during the procedure resulting in increased chronic kidney disease stage or the need for dialysis [11]. Consequently, it is discussed that percutaneous image-guided cryoablation could play a role in treating small RCCs and preserving healthy renal parenchyma.

The aim of this multicentre cohort study was to explore how renal function was affected after percutaneous image-guided cryoablation in patients with solitary kidney presented with RCC.

## Methods and Materials

### Study Design

The study was conducted, and the manuscript was prepared according to the Strengthening the Reporting of Observational Studies in Epidemiology (STROBE guidelines) [12]. The study cohort of patients was prospectively included in the European Registry for Renal Cryoablation (EuRECA) database from 2014 until present. The database and study are done in accordance with the principle of the Helsinki Declaration and fulfil the European General Data Protection Regulation (GDPR) rules.

### Setting

The EuRECA registry is an international multicentre collaboration including 16 centres from seven European countries with the overall aim of prospectively gathering information and knowledge about image-guided and surgical cryoablation for small renal tumours [13]. The registry covers a period from 2014 to 2020 with a total of 1672 patients registered. In the present study, we aimed to analyse a subgroup of these patients with solitary kidneys.

### Participants

The aim of the study was to explore how renal function was affected after one percutaneous image-guided cryoablation in patients with solitary kidney and RCC. Thereby, patients treated more than one time within the three months or for more than one tumour were not included.

Participants were eligible for inclusion if they:

- Were treated with image-guided percutaneous cryoablation for RCC in a solitary kidney at one of the collaborative centres, AND
- Present baseline and at least one post-operative estimated glomerular filtration rate (eGFR) measurement (closest to three-month follow-up ± one month), AND
-  > 18 years old.

Patients were excluded from the study if they:

- Had more than one renal tumour detected and/or treated, OR
- Had undergone cryoablation, partial nephrectomy, or other treatment modalities for the current or previous renal tumour in the solitary kidney, OR
- Were treated with more than one cryoablation procedure.

### Variables, Data Source, and Measurements

Data on the following variables were collected from the patients and procedures: patient demographics (age, gender, weight, height, Charlson Comorbidity Score [14], American Society of Anesthesiology (ASA) score), tumour characteristics (RENAL nephrometry score [15], pre-operative histology), and renal function measures (eGFR based on creatinine levels using the Modified of Diet in Renal Disease (MDRD) equation, presented in unit: mL/min/1.73 m^2^). Some patients had several renal function measurements after the procedure. Post-ablation renal function outcomes were defined in this study as the measurement of creatinine level and thereby eGFR calculation closest to three months after the procedure but not before two months and not exceeding four months. Procedure-related data were also extracted (anaesthesia, number of cryoablation needles and type, number of freeze–thaw cycles, as well as perioperative and post-operative complications using the Clavien–Dindo score [16].

### Statistical Methods

Descriptive statistics were performed for each variable. Categorical variables were presented with frequencies and percentages, and continuous variables were presented with the median and interquartile range (IQR). The primary outcome was change in renal function (defined as mean eGFR in mL/min/1.73 m^2^ and eGFR group) from baseline to follow-up (closest to 3 months ± 4 weeks), using paired t-test and Wilcoxon signed-rank test, respectively. A *p*-value less than 0.05 was considered statistically significant, and no correction for multiple testing was utilized. The STATA 17 software (StataCorp, 2021, Stata Statistical Software: Release 17, College Station, TX, StataCorp, LLC) was used to perform the statistical analyses.

## Results

### Patients

**Fig. 1:**
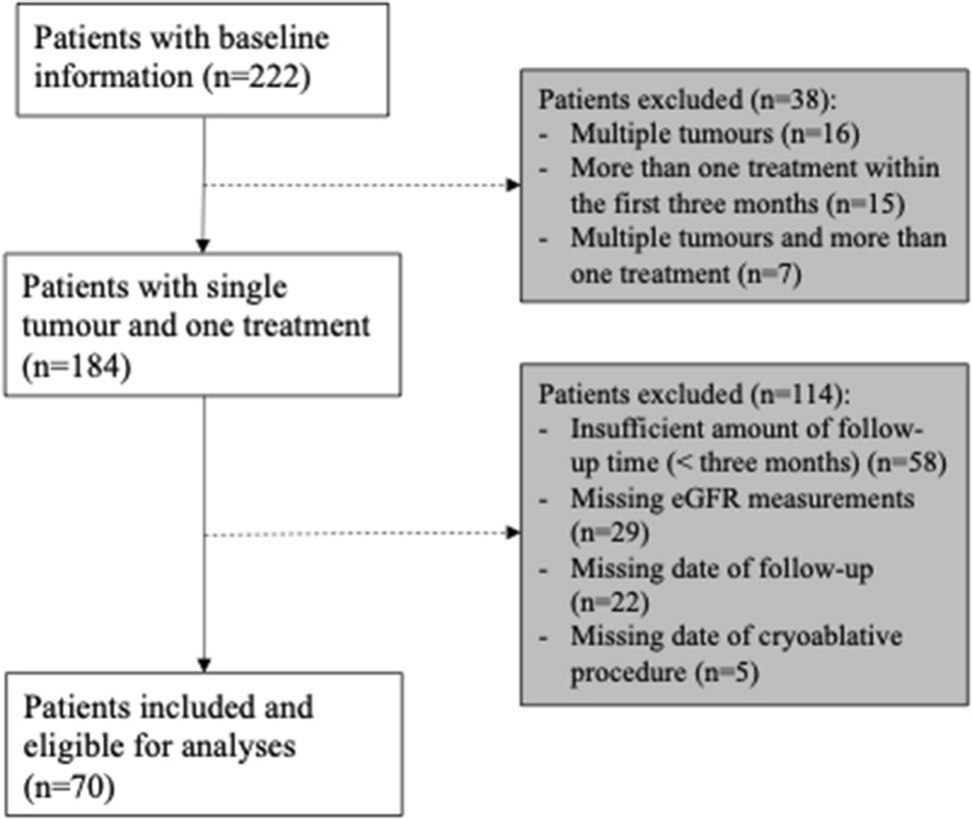
Patient flowchart

A total of 222 patients with solitary kidney and biopsied confirmed renal cancer were entered into the database. Of these, 184 patients were included following the inclusion criteria, and 70 patients (38%) had sufficient available data on baseline and follow-up examinations and were included in the analysis, see Fig. 1.

Patient demographics and tumour characteristics are presented in Table 1. The median age was 67.5 [IQR 61.0–75.0], and the majority of the patients were men (women *n* = 23, 32.9%, and men *n* = 47, 67.1%). The RENAL nephrometry score was available for all patients with a median of 6.0 [IQR 4.0–7.0].

**Table 1:** Patient characteristics

Age, median [IQR]	67.5 [61.0–75.0]
Gender, *n* (%)	
Men	47 (67.1)
Women	23 (32.9)
Charlson comorbidity index, median [IQR]	2.5 [2.0–4.0]
RENAL nephrometry score, median [IQR]	6.0 [4.0–7.0]
Size in cm, *n* (%)	
< 4	22 (31.4)
4–7	19 (27.1)
< 4	29 (41.4)
Exophytic/endophytic, *n* (%)	
> 50% Exophytic	18 (25.7)
< 50% Exophytic	35 (50.0)
Entirely endophytic	17 (24.3)
Nearness to collecting system or sinus in mm, *n* (%)	
> 7	22 (31.4)
4–7	19 (27.1)
< 4	29 (41.4)
Location, *n* (%)	
Anterior	24 (34.3)
Posterior	24 (34.3)
Neither	22 (31.4)
Location relative to polar lines, *n* (%)	
Entirely above or below	35 (50.0)
Cross a polar line for < 50%	12 (17.1)
Cross a polar line for > 50%; lesion crosses the axial line; lesion is between polar lines	23 (32.9)
Hilar tumour, *n* (%)	
Yes	6 (8.6)
No	64 (91.4)

*IQR* Interquartile range

**Fig. 2:**
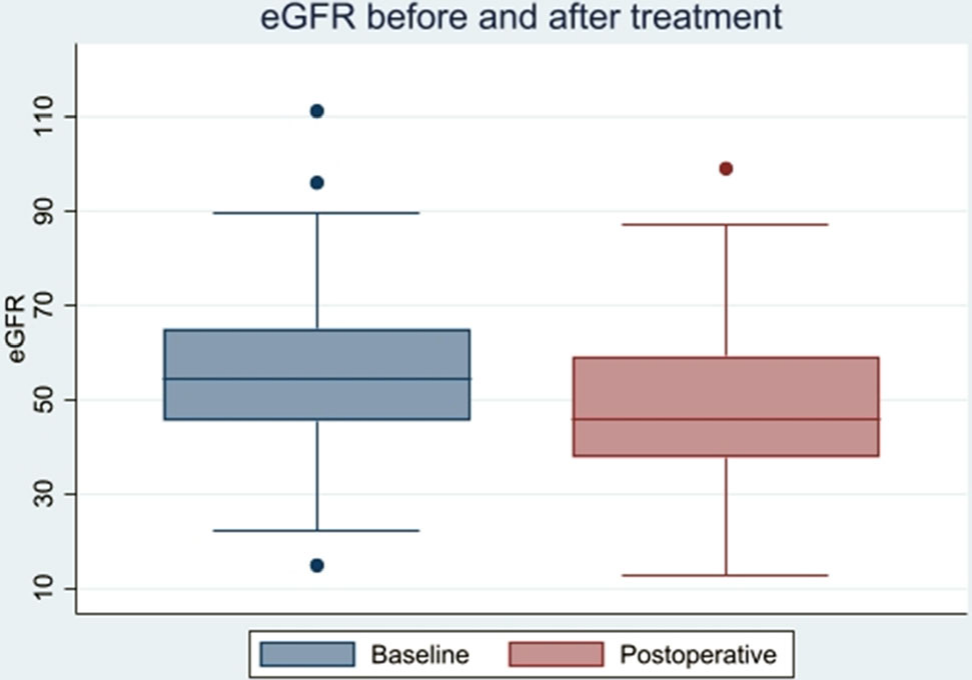
Boxplot of eGFR before and after treatment

The baseline and post-operative eGFR data are presented in Table 2 and as boxplots in Fig. 2. The mean baseline eGFR was 55.8 ± 16.8 mL/min/1.73 m^2^, and the mean 3-month post-operative eGFR was 49.6 ± 16.5 mL/min/1.73 m^2^, which led to a mean eGFR reduction at − 6.2 mL/min/1.73 m^2^, amounting a percentage reduction of 11.1% (*p* ≤ 0.01). Table 2 also includes the number of patients in the stages of chronic kidney disease and reveals a general advancement of patients increasing from chronic kidney disease I and II to IIIa and IIIb, but none of the patients advanced to category V (end-stage chronic kidney disease) and only one patient from stage IIIb to IV.

**Table 2:** Estimated glomerular filtration (eGFR) rates at baseline and follow-up

	Baseline	3 months	Mean difference (95% CI)	*p*-value
eGFR in mL/min/1.73 m^2^, mean [SD]^a^	55.8 [16.8]	49.6 [16.5]	− 6.2 (− 8.2; − 4.2)	< 0.001*
Chronic kidney disease stage, *n* (%)^b^				< 0.001*
I	2 (2.9)	1 (1.4)	N/A	
II	25 (35.7)	16 (22.9)		
IIIa	26 (37.1)	19 (27.1)		
IIIb	14 (20.0)	30 (42.9)		
IV	2 (2.9)	3 (4.3)		
V	1 (1.4)	1 (1.4)		

^a^Paired *t*-test for the mean difference between baseline and 3-month eGFR. Normality assessed via visualizations of the data and Shapiro–Wilk *W* test for normality

^b^Wilcoxon signed-rank test for equality between eGFR groups at baseline and 3 months

^*^p < 0.05, statistical significance

## Discussion

Renal function reduction following image-guided percutaneous cryoablation for RCC in patients with solitary kidneys is low. The treatment does not entail an acute need for dialysis or severe advancement in chronic kidney disease stage despite a statistically significant 11% reduction in renal function. Whether an 11% reduction is clinically relevant with the severe cancer diagnosis in mind is debatable and depends on each specific patient.

Guidelines describe to varying degrees the use of cryoablation for renal cell carcinoma. The 2022 guideline by the European Association of Urology (EAU) confirms that cryoablation—independent of a laparoscopic or CT-guided percutaneously approach—has a high technical success and low complication rates [17]. A meta-analysis by Zargar et al. [18] concludes that cryoablation has a technical success at > 95% and complication rates between 8 and 20%. In regard to oncological outcomes, for T1a tumours, the long-term oncological outcomes following image-guided ablative therapy are similar to surgical intervention. For long-term oncological outcomes of T1b tumours, the conclusion of the meta-analysis was not definite. Cancer-specific survival was similar (based on seven studies); however, in one matched cohort the cancer-specific survival was significantly worse for patients undergoing ablative therapy. The meta-analysis concludes that a number of factors, including the relatively small size of the studies, single-centre setting, and operator competence variation across institutions, contribute to uncertainty about the results and conclusions and that larger, controlled multicentre studies are needed in order to resolve the uncertainty [7].

In regard to cryoablation and renal function outcomes, the EAU guidelines refer to a cohort study by Pickersgill et al. [19] enrolling 308 patients undergoing CT-guided cryoablation for small renal masses. Pickersgill et al. [19] presented an eGFR reduction after cryoablation treatment at approximately 11% which is the same reduction as in the current study—but in a patient group with two kidneys. A propensity score-matched study by Bhindi et al. [20] explored the differences between partial nephrectomy and CT-guided cryoablation in patients with RCC and solitary kidney. The study included 54 patients receiving cryoablation and 64 receiving laparoscopic partial nephrectomy. Their study cohort had, as in the current study, a mean baseline renal function at 56 mL/min/1.73 m^2^ for both groups and an 11% renal function reduction in the cryoablation group at discharge and a 12% reduction after three months, which corresponds to our cohort. When comparing the renal function reduction between cryoablation and partial nephrectomy, there was no significant difference.

While surgical and ablative management of small RCC are well established, another option for this subgroup of patients is active surveillance or watchful waiting. The clear advantage of active surveillance or watchful waiting is to postpone or avoid intervention with potential complications, especially the initiation of dialysis [21]. The disadvantages include the risk of local or distant progression of the disease and the potential requirement for a larger surgical or ablative procedure. In patients with a solitary kidney, this option remains a management option, dependent on factors such as patient age, frailty, comorbidity, and renal function, as well as tumour size and growth characteristics. Every case should be discussed in a multidisciplinary team meeting.

The literature of active surveillance for patients with tumours in solitary kidneys is limited, but that it should be considered as an option for especially elderly and fragile patients—the same patient population that are eligible for image-guided cryoablation.

Thus, in patients with solitary kidney and RCC, several variables are to be included in the decision-making on management and potential treatment. The complexity of renal cancer treatment increases, and the advantages and disadvantages of each treatment should be considered. Factors like quality of life, adverse events, or side effects of focal versus systemic therapy must be discussed with the patient and relatives.

A systematic review on quality of life after partial nephrectomy or cryoablation of T1 RCC in patients with two kidneys concluded that nephron-sparing surgery appears to be either superior or comparable to other treatment alternatives concerning quality of life outcomes [22]. In a prospective study by Junker et al. including 165 patients undergoing partial nephrectomy or image-guided cryoablation, they found a significant difference between baseline and 14-day follow-up in several quality of life and symptoms scales, favouring cryoablation over partial nephrectomy [23]. However, no significant differences were observed in any quality of life scales after 90-day follow-up. In the study, patients receiving cryoablation were significantly older and had lower scores on physical and role functioning than those undergoing partial nephrectomy. A follow-up (NEST-2) of the recent randomized controlled feasibility study, including 200 patients randomized for either cryoablation or robotic partial nephrectomy, is on the horizon to address many of these points raised [24].

The European collaboration of the EuRECA database increases the generalizability and external validity, and data thereby do not reflect technique, variables, or bias from a single centre. Since it is a registry rather than a prospective study, each centre carried out follow-up according to local practice. In addition, many of the contributors work at tertiary centres where patients may not return routinely for a face-to-face consultation (and therefore a blood test on the local laboratory system), especially after March 2020 when the COVID-19 pandemic reduced this further.

The data were collected prospectively, but several limitations exist. First, we acknowledge the amount of missing data as being significant. Many patients were excluded from the study due to insufficient or missing follow-up. Because we wanted to explore the change in renal function approximately three months after the image-guided cryoablation procedure, all patients without precise dates for blood tests were excluded. The different and dynamic follow-up regimes in the different countries and institutions did not allow us to collect the missing data retrospectively. Secondly, the study was non-controlled and non-randomized, and therefore, a selection bias of the patients included is probable. In the original protocol, it was the intention to include a secondary outcome exploring the relationship and correlation between the reduction in renal function and variables like tumour size and nephrometry score. Due to the significant amount of missing data on several of the included patients, the study was not powered to make conclusions on this. The tumours were most often small (< 4 cm), only with a partial exophytic component, and not close to the renal hilum, as reflected in the RENAL nephrometry scores. Consequently, the tumours may not be representative of all renal tumours in solitary kidney patients.

The aim of the study was not to explore the differences in renal function reduction after image-guided cryoablation versus partial nephrectomy, but to *estimate* the reduction following cryoablation in patients with a solitary kidney and a single small RCC. The results add information and evidence so that oncologists, surgeons, and interventional radiologists in the future, and as a multidisciplinary team decision, can discuss the advantages and disadvantages of cryoablation for patients with solitary kidneys.

In conclusion, this study found that patients undergoing percutaneous image-guided cryoablation for small renal tumours have a high preservation of renal function. Despite a statistical decrease in the renal function of 11% after 3 months (55.8 ± 16.8 vs. 49.6 ± 16.5 mL/min/1.73 m^2^), all patients avoided the need for acute dialysis, and none had a severe advancement in chronic kidney disease stage. This treatment should be considered as an opportunity for patients with small renal tumours and solitary kidneys.
